# Empirical treatment and mortality in bacteremia due to extended spectrum β-lactamase producing Enterobacterales (ESβL-E), a retrospective cross-sectional study in a tertiary referral hospital from Colombia

**DOI:** 10.1186/s12941-023-00566-2

**Published:** 2023-02-16

**Authors:** Deving Arias Ramos, John Alexander Alzate, Germán Alberto Moreno Gómez, Julián Andrés Hoyos Pulgarín, Juan Camilo Olaya Gómez, Isabella Cortés Bonilla, Camila Vargas Mosquera

**Affiliations:** 1 Universidad Tecnológica de Pereira, Pereira, Colombia; 2 Hospital Universitario San Jorge, Pereira, Universidad Tecnológica de Pereira, Pereira, Colombia; 3 Oncólogos de Occidente, Pereira, Colombia; 4 Grupo de investigación en Medicina Interna, Universidad Tecnológica de Pereira, Pereira, Colombia

**Keywords:** Bacteremia, Antimicrobial resistance, Extended spectrum β-lactamase (ESβL)

## Abstract

**Background:**

Infections caused by extended spectrum β-lactamase (ESβL) producing bacteria are common and problematic. When they cause bloodstream infections, they are associated with significant morbidity and mortality.

**Methods:**

A retrospective cross-sectional observational study was conducted in a single center in Pereira, Colombia. It included people hospitalized with bacteremia due to gram-negative bacilli with the extended-spectrum β-lactamase producing phenotype. A logistic regression analysis was constructed. Clinical characteristics and risk factors for death from sepsis were established.

**Results:**

The prevalence of bacteremia due to Enterobacterales with extended-spectrum β-lactamase producing phenotype was 17%. 110 patients were analyzed. Most patients were men (62%) with a median age of 58 years, hospital mortality was 38%. Admission to intensive care was 45%. The following risk factors for mortality were established: shock requiring vasoactive support, Pitt score > 3 points, and not having an infectious disease consultation (IDC).

**Conclusions:**

bacteremia due to Enterobacterales with extended-spectrum β-lactamase producing phenotype have a high mortality. Early recognition of sepsis, identification of risk factors for antimicrobial resistance, and prompt initiation of appropriate empiric antibiotic treatment are important. An infectious disease consultation may help improve outcomes.

## Background

Resistance in gram-negative bacteria has gained great importance in recent decades and one of the reasons is the increase in extended-spectrum β-lactamase-producing bacteria (ESβL) as a growing problem throughout the world [1–4]. ESβL are β-lactamases that can hydrolyze penicillins and cephalosporins [5]. High prevalence rates of ESβL-producing Enterobacterales (ESβL-E) have been reported in *Escherichia coli* and *Klebsiella pneumoniae* [6].

The prevalence of ESβL-producing *K. pneumoniae* and *E. coli* have steadily increased over the last decades. ESβL expression by Enterobacterales in Latin American hospitals became endemic since 1990 [7]. In 2002, CTX-M-12 from a strain of *K. pneumoniae* was detected in Colombia, representing the first description of a CTX-M in our country [8]. Currently, hospitals in Latin America face the problem of high infection rates by ESβL-producing Enterobacterales. It is important to note that ESβL-E infections are associated with higher mortality (up to 70%) compared to patients with bacterial infections without production of ESβL-E [9]. Patients have a survival advantage when the correct antimicrobial agent is chosen as initial therapy [7].

The aim of the study was to describe the clinical characteristics of hospitalized patients with ESβL-E bacteremia. This was a single center study, developed at the *Hospital Universitario San Jorge*, from the city of Pereira, in Colombia. Pereira is one of the main cities of the coffee region, with a population of more than 500,000 inhabitants.

## Methods

### Study design and data collection

This was a retrospective, cross-sectional, observational study conducted at the *Hospital Universitario San Jorge de Pereira*, Colombia, a tertiary referral center with more than 300 beds, located in the city of Pereira, Colombia. The study was approved by the Ethics Committee of the *Universidad Tecnológica de Pereira*. The study period was from January 2012 to December 2017. All patients with ESβL-E bacteremia were included. Those patients with incomplete clinical records or those who did not die from sepsis were excluded. Electronic medical records were reviewed. Data on the clinical characteristics of the patients, comorbidities, symptoms, and laboratory tests at hospital admission were collected. A logistic regression analysis was constructed. Risk factors for sepsis mortality were established.

### Definitions

Hospital-acquired bacteremia was considered if the blood culture (BC) sample was taken 48 h after hospital admission [10]. The criteria of Friedman et al. were used to determine which infections were healthcare associated [11]. Infections acquired in the community were considered when the first positive culture was obtained < 48 h from the patient's admission to the hospital [12]. Primary bacteremia was defined as bacteremia for which no source of infection was documented [12]. Secondary bacteremia was considered when a source of infection was found, at the same time or up to 3 days before the bacteremia [12]. The source of the bacteremia was confirmed by the clinical characteristics of each patient, the clinical evaluation performed by the physician, and the reports of cultures taken from other anatomic sites. Catheter related bloodstream infection (CRBSI) was defined based on the positivity time of the BCs obtained at the same time, one from the catheter and the other from peripheral venipuncture, processed in a continuous monitoring system. If both BCs grow the same organism and the BC drawn from the device becomes positive > 2 h before the BC drawn by venipuncture, CRBSI was considered [13].

Febrile neutropenia was considered when the neutrophil count was < 500 cells/mm^3^ [14]. The Charlson comorbidity index was used to assess the impact of comorbidities in the study population. Sepsis and septic shock were defined according to The Third International Consensus Definitions for Sepsis and Septic Shock (Sepsis-3) [15]. Mortality was defined as inpatient death due to bacterial sepsis. Only mortality attributed to sepsis was taken into account. Complete medical records were reviewed to establish the cause of death. All patients with septic shock and/or acute respiratory failure were considered for admission to critical care.

Empiric antibiotic therapy consisted of antibiotics administered within 24 h of identifying a case of bacterial sepsis. Definitive therapy was defined as antibiotics instituted after the results of the blood culture and antibiogram were known. Empirical treatment was considered adequate when a carbapenem was administered, any other β-lactam antibiotic was considered inappropriate regardless of in vitro susceptibility. The dose, frequency, and route of antibiotics were not taken into account when assessing suitability. All study definitions were established prior to data analysis.

### Laboratory methods

The samples analyzed included blood, urine, and respiratory tract samples (sputum expectorated by the patient or orotracheal tube aspirate). The detection of ESβL was performed by an automated method. The Vitek 2 system (bioMérieux SA) was used. All isolates showed sensitivity to carbapenems according to the CLSI (Clinical & Laboratory Standards Institute) cut-off points for each year.

### Collection and processing of blood samples

The volume of blood that was obtained, for adults, was 20–30 mL of blood per culture set, > 2 culture bottles were used. For neonates and adolescents, an age- and weight- appropriate volume of blood was cultured according to guidelines [13]. Inoculated blood culture bottles were incubated at 37 °C and inspected for 5 consecutive days [16]. Blood cultures that showed signs of bacterial growth were subcultured on blood agar and MacConkey agar [16]. Agar plates were incubated at 37 °C for 48 h. Gram-negative rod colonies were pre-identified by their colony appearance and morphology on Gram stain [16]. All isolates were confirmed by the automated Vitek2 system (Biomerieux, France).

Laboratory tests included complete blood count, liver function tests, renal function tests, urinalysis, urine gram stain, urine culture. Chest X-rays, computed tomography (CT) of the chest, and CT of the abdomen were performed when deemed necessary.

### Statistic analysis

Descriptive statistics were calculated. Normality was assessed using the Kolmogorov Smirnov test or the Shapiro Wilk test when appropriate. For the continuous variables, the assumptions of normality were verified and for those that met them, Student’s t tests were performed. Nonparametric tests were used for those that did not meet the assumptions of normality (Wilcoxon or Man Whitney U). Continuous variables were presented as mean ± standard deviation or as medians and interquartile ranges (IQRs) when appropriate. Categorical variables were presented as frequency and percentage. Categorical variables were analyzed using the Chi-square test or Fisher’s exact test when appropriate. Variables with a p value < 0.1 on bivariate analysis were then entered into a logistic regression model to identify risk factors for death from sepsis. A stepwise logistic regression was constructed. A value of p < 0.05 was considered as the cut-off point for statistical significance. The odds ratio was calculated with 95% confidence intervals.

The Hosmer and Lemeshow test was used to verify compliance with the necessary assumptions for the implementation of logistic regression, a value of p > 0.005 was considered a good fit.

IBM SPSS Statistics version 20 software was used for all statistical analyses.

## Results

**Fig. 1:**
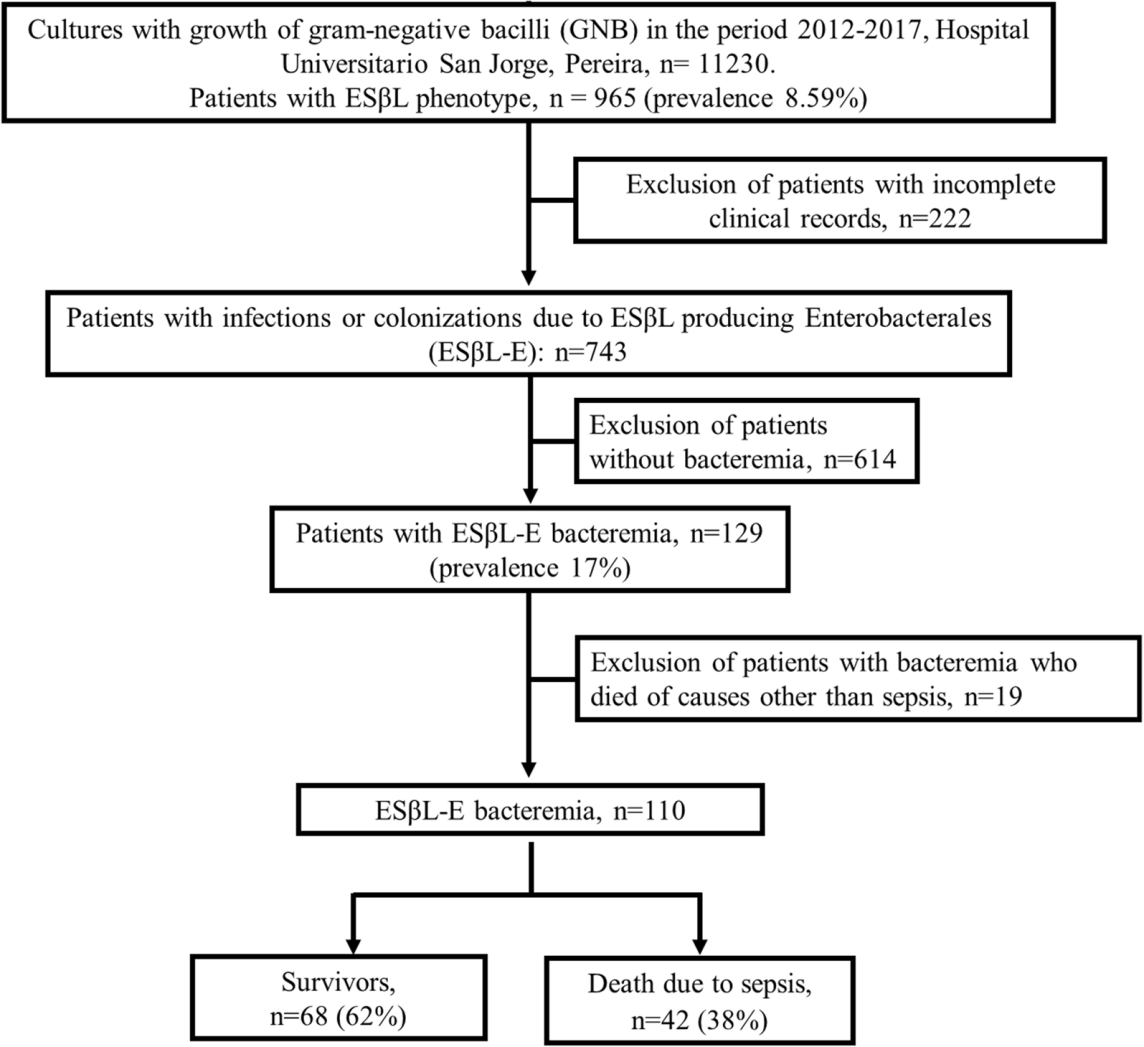
Flowchart for patient selection

110 patients were included. Figure 1 shows the flowchart for patient selection. The median age was 58 years, with an interquartile range (IQR) of 35 to 73, only 14 patients were < 18 years old, 62% (n = 69) were men, and 40% (n = 45) were from rural areas. Hospital mortality was 38% (n = 42). The most frequent comorbidities were high blood pressure (48%), cancer (28%), type 2 diabetes mellitus (24%) and chronic obstructive pulmonary disease (COPD) (19%). See Table 1.

**Table 1:** Clinical characteristics

Variable	All patients n = 110 (%)	Survivors n = 68 (%)	Death due to sepsis n = 42 (%)	P	OR	CI95%
Age in years, median (IQR)	58 (35–73)	58 (34–73)	60 (35–73)	0.7		
Men	69 (62)	45 (66)	24 (57)	0.3		
Days of hospital stay, median (IQR)	26 (13–46)	32 (18–56)	21 (9–34)	0.001		
Critical care admission	50 (45)	24 (35)	26 (61)	0.006	2.9	1.3–6.6
Ventilatory support	41 (37)	13 (19)	28 (66)	0.001	8.4	3.5–20
Days in critical care, median (IQR)	8 (3–20)	8 (4–17)	7 (1–24)	0.3		
Serum creatinine, mg/dL, median (IQR)	0.8 (0.6–1.2)	0.8 (0.6–1.2)	0.8 (0.6–1.3)	0.3		
Acute kidney injury	41/101 (40)	24/61 (39)	17/40 (42)	0.7		
Serum lactate levels, mmol/L, median (IQR)	2 (1–3)	1.2 (1–3)	2 (1–2.9)	0.5		
Lactate levels ≥ 2 mmol/L	36/70 (51)	16/37 (43)	20/33 (60)	0.1		
Shock requiring vasopressor support	45 (40)	18 (26)	27 (64)	0.001	5	2.1–11.4
Pitt bacteremia score, median (IQR)	2 (0–4)	2 (0–2)	3 (1.7–4)	0.001		
Pitt bacteremia score 0–1 point	43 (39)	33 (48)	10 (23)	0.01	0.3	0.1–0.7
Pitt bacteremia score > 3 points	28 (25)	9 (13)	19 (45)	0.001	5.4	2.1–13.6
qSOFA at the time sepsis is suspected, 2–3 points	41 (37)	22 (32)	19 (45)	0.1		
Charlson comorbidity index, median (IQR)	4 (2–6)	4 (2–6)	4 (1.7–6.2)	0.9		
Charlson > 4 points	48 (43)	29 (42)	19 (45)	0.7		
Community acquired infection	28 (25)	19 (27)	9 (21)	0.4		
Hospital-acquired infection	82 (74)	49 (72)	33 (78)	0.4		
Presence of risk factors for healthcare-associated infection	84 (76)	48 (70)	36 (85)	0.07		
Secondary bacteremia	86 (78)	53 (77)	33 (78)	0.9		
Primary bacteremia	13 (11)	8 (11)	5 (11)	0.9		
Catheter-associated bloodstream infection	11 (10)	7 (10)	4 (9)	0.8		
Urinary tract infection	25/86 (29)	20/53 (37)	5/33 (15)	0.02	0.29	0.09–0.8
Source of infection other than urinary tract	61/97 (62)	33/60 (55)	28/37 (75)	0.04	2.5	1.02–6.3
Pneumonia	18/86 (20)	8/53 (15)	10/33 (30)	0.09		
Febrile neutropenia	12/86 (14)	8/53 (15)	4/33 (12)	0.7		
Sepsis of abdominal origin (peritonitis, cholangitis)	30/86 (34)	17/53 (32)	13/33 (39)	0.4		
High blood pressure	53 (48)	28 (41)	25 (59)	0.06		
Diabetes mellitus type 2	27 (24)	16 (23)	11 (26)	0.7		
Cancer	31 (28)	19 (27)	12 (28)	0.9		
COPD	21 (19)	12 (17)	9 (21)	0.6		
Chronic kidney disease stage 4 and 5	19 (17)	9 (13)	10 (23)	0.1		
Heart failure	19 (17)	12 (17)	7 (16)	0.8		
Empirical antibiotic therapy	104 (94)	65 (95)	39 (92)	0.5		
Total days of empirical treatment (appropriate and inappropriate)	3 (2–5)	3 (2–5)	3 (2–4)	0.5		
Empirical treatment with carbapenem (appropriate treatment)	44/104 (42)	27/65 (41)	17/39 (43)	0.8		
Empirical treatment with non-carbapenem antibiotics	60/104 (57)	38/65 (58)	22/39 (56)	0.8		
Ampicillin sulbactam	12/104 (11)	8/65 (12)	4/39 (10)	1		
Piperacillin tazobactam	29/104 (27)	18/65 (27)	11/39 (28)	0.9		
Cefepime	14/104 (13)	10/65 (15)	4/39 (10)	0.4		

Primary bacteremias were 11%, CRBSI were 10%, and secondary bacteremias were 78%. The most common source of infection in secondary bacteremia was abdominal sepsis in 34% (due to peritonitis in 5 patients and cholangitis in 25 patients), urinary tract infection in 29%, pneumonia in 20%, and febrile neutropenia in 14%. Urinary tract infection was more common in the surviving group of patients (37% vs 16%, p = 0.02). Death was more common when the source of infection was not from a urinary tract infection (75% vs 55%, p = 0.04). Bladder catheterization was very common (41%) and the prevalence of biliary stents was 19%.

The population served was quite ill. The Charlson comorbidity index had a median of 4 points (IQR of 2–6 points). Admission to critical care was high (45%) and ventilatory support was provided in 37%. In the population that survived a Pitt score of 0–1 points was found more frequently compared to the group that died of sepsis (48% vs 23%, p = 0.01), on the other hand, a Pitt score > 3 points was more common in the group that died from sepsis (45% vs 13%, p = 0.001). There was no difference between the groups in the abnormal qSOFA scale (> 1 point). See Table 1.

The hospital stay was prolonged (median 26 days, IQR 13–46), also, the days of stay in intensive care were prolonged (median 8 days, IQR 3–20). The creatinine value at admission was 0.8 mg (IQR 0.6–1.2), 40% developed acute kidney injury. There was no difference between groups in the incidence of acute kidney injury. There were also no differences between groups in lactate levels.

### Antibiotic treatment

**Fig. 2:**
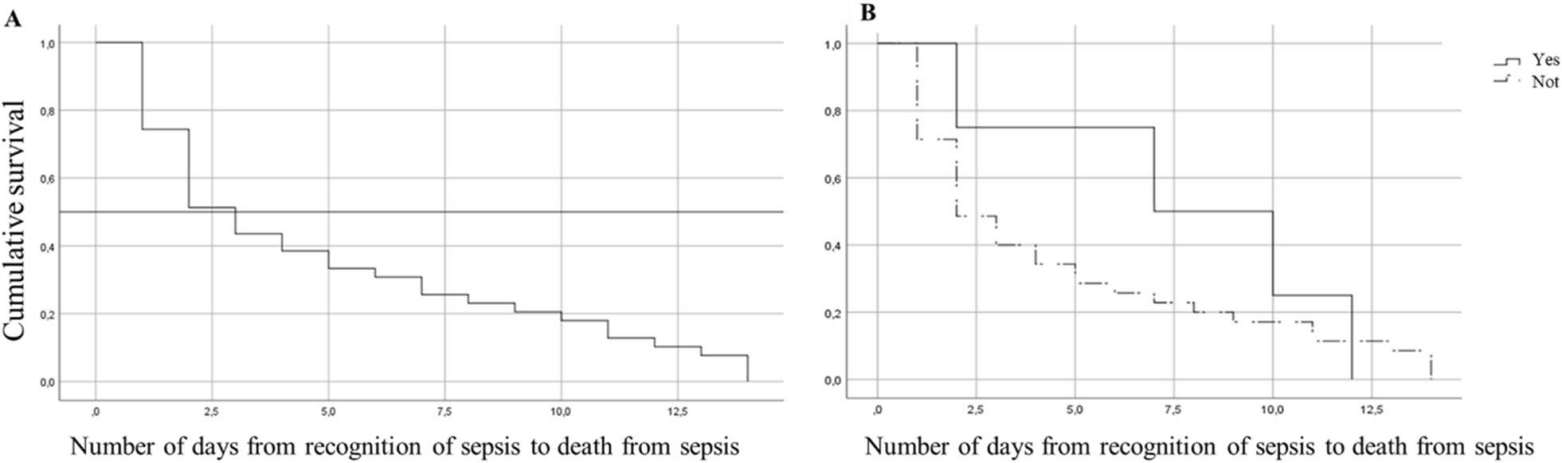
**A** Shows the cumulative survival of the total population, 52% of deaths occurred in the first 3 days from the recognition of sepsis. **B** Shows the cumulative survival of patients who had an evaluation by an infectious disease specialist, compared to those who did not

Most patients received empirical treatment. There were 6 patients (5.4%) who did not receive empirical antibiotic treatment. Empirical treatment was appropriate in 42% (n = 44/104). The remaining 60% (n = 66/110) of the population were amenable to carbapenem treatment (appropriate treatment) when culture results (susceptibility testing) were available. There were no differences between groups in the proportion of patients who received empirical treatment (95% vs 92%, p = 0.6), nor in the adequacy of treatment (whether or not they received a carbapenem) (41% vs 43%, p = 0.8). The most empirically used non-carbapenem β-lactam antibiotics were piperacillin tazobactam (27%), cefepime (13%) and ampicillin-sulbactam (11%). See Table 1. The median number of days from recognition of sepsis to death from sepsis was 3 days (IQR 1–8). About 52% of deaths occurred in the first 3 days from recognition of sepsis. Empirical treatment was given for a median of 3 days (IQR 2–5 days). See Fig. 2. Death was less common in those who completed > 7 days of carbapenem treatment.

Targeted therapy (based on the result of the antibiogram) was possible in 77% of patients (n = 51/66) who were amenable to carbapenem treatment when the antibiogram was finally known.

When the results of blood cultures with antibiograms were known, it was more common to receive antibiotic treatment with a carbapenem in the group of patients who survived compared to the group who died of sepsis (95% vs 48%, p = 0.001, OR 0.04, 95% CI 0.01–0.24). This finding firstly suggested a case of therapeutic inertia for antibiotic prescription based on the interpretative reading of the antibiogram. However, the data show that in the group of patients who died from sepsis (n = 42/110), 59.5% (n = 25/42) of the patients did not receive empirical carbapenem treatment (appropriate treatment) and 48% (n = 12/25) of them died in the first 72 h from the recognition of sepsis, therefore, doctors did not get to know the results of blood cultures while the patients were still alive. Figure 2 shows how most deaths occurred in the first 3 days after recognition of sepsis.

70% (n = 77/110) of the patients did not receive an infectious disease consultation (IDC). Absence of IDC was more common in the group that died from sepsis compared with those that survived (85% vs 60%, P = 0.005, OR 3.9, 95% CI 1.4–10.6). Follow-up blood cultures (FUBC) were taken in 23 patients (21%), 34% (n = 8/23) showed persistent bacterial growth.

*Klebsiella pneumoniae* was identified in 51% *and Escherichia coli* in 49%. The minimum inhibitory concentration (MIC) of ciprofloxacin had a median of 4 µg/mL (IQR 4–4), the MIC of gentamicin had a median of 1 µg/mL (IQR 1–16), and the MIC of amikacin had a median 3 µg/mL (IQR 2–8). No Clostridium difficile infection was diagnosed, but this was not routinely screened for in the patients.

### On the use of piperacillin tazobactam

The MIC of piperacillin-tazobactam had a median of 16 µg/mL (IQR 8–64), mean of 42 µg/mL. 27.8% (n = 29/104) of the patients had empirical treatment with Piperacillin tazobactam, later 75% (n = 22/29) were escalated to carbapenems when the results of the blood cultures were known, 20.6% (n = 6/29) died in the first 72 h, before knowing the result of the antibiogram. The mortality of the group that received piperacillin tazobactam as empirical treatment was 37.9% (n = 11/29).

### Logistic regression

The stepwise logistic regression analysis showed that the variables associated with death from sepsis are a Pitt score > 3 points, a state of septic shock requiring vasoactive support and not received an IDC. See Table 2.

**Table 2:** Logistic regression, risk factors for in-hospital death

Variable	OR	P-value	95% CI
Pitt bacteremia score > 3 points	4.094	0.016	1.30–12.85
Not receiving an infectious disease consultation	5.436	0.004	1.71–17.23
Shock requiring vasopressor support	3.171	0.020	1.20–8.37

In the logistic regression analysis, the Omnibus test of the coefficient model had a P value of 0.001; Nagelkerke’s R was 32.5% (0.325); the Hosmer and Lemeshow test showed a P value of 0.883

## Discussion

The present study described the clinical characteristics and risk factors for death from ESβL-E bloodstream infections (BSI). The population was quite morbid, we found high rates of admission to critical care, presence of renal failure at admission, and requirement of ventilatory support. The mortality in our study was very high. Many scientific publications have reported different levels of mortality in BSI caused by ESβL-E. In most reports, mortality is high, but with a wide range, for example, Lim et al. reported a 20% mortality [10] but, Namikawa et al. reported a 9.7% mortality [17], Scheuerman et al. reported an ESβL-producing *K. pneumoniae* BSI mortality of 33% and, an ESβL-producing *E. coli* BSI mortality of 17% [18], also, Tuon et al. reported a mortality from ESβL-producing *K. pneumoniae* of 49% [19]. The enormous heterogeneity that exists between the characteristics of the population of each mortality report may explains this notable variation in mortality from ESβL.

In considering the explanation of the high mortality found, we found no differences in mortality due to sepsis regarding the source of infection (primary bacteremia, secondary bacteremia, or CRBSI), the qSOFA, the initial lactate value and, the Charlson score. We found no differences between groups regarding the empirical administration of treatment with a carbapenem (41% vs 43%, p = 0.8), data similar to those shown by Lim et al. [10], who also analyzed the impact of empiric treatment in similar infections. In our cohort, there is a mortality peak in the first 3 days after recognition of sepsis (see Fig. 2). We observed that a high percentage of patients did not receive treatment based on the antibiogram. This was mainly observed in patients who died within the first 3 days from recognition of sepsis. These results would favor the use of rapid molecular diagnostic tests based on positive blood cultures to strengthen the appropriate prescription of antibiotics in bacteremia [20]. However, the population that died was more severely ill and probably benefited more from received empiric carbapenem therapy since initial evaluation. This is evidence of a poor perception of the risk of ESβL-E infection by clinicians when prescribing empirical antibiotic treatment.

In our cohort there was a high prevalence of risk factors for healthcare-associated infections and the group that died due to sepsis was more compromised in their health status: they were most commonly affected by septic shock, they had higher admission to critical care, they had higher requirement of ventilatory support and they had higher Pitt bacteremia score; therefore, based on current recommendations [21, 22], these patients should have received a higher proportion of treatment with carbapenems, however only 43% of this group received it. Similar findings were reported by Tuon et al., where the authors found that only 52% received adequate treatment within 48 h of bacteremia [19].

An IDC may play a role that can impact the outcome of mortality. This finding has been shown in bacteremia due to *Staphylococcus aureus* [23] and now we observe it in ESβL-E bacteremia (see Fig. 2). In the case *S. aureus* bacteremia, in the study of Bai et al. [24], it was shown that patients who received an IDC were more likely to have an echocardiogram, FUBCs, receive appropriate empiric antibiotic treatment and, have a longer duration of antibiotic therapy. As we know, there is no evidence on the benefit of an echocardiography in Gram-negative bacterial bloodstream infection caused by ESβL-E. Echocardiogram should be performed for infections caused by the HACEK gram-negative bacteria, but those are very rare infections [25, 26].

One of the reasons why an IDC could reduce the in-hospital mortality due to sepsis is due to the appropriate selection of empiric antibiotic treatment from the moment sepsis was first suspected or recognized. In our study, 51% (n = 17/33) of the patients who had an IDC had appropriate empirical antibiotic treatment with carbapenems, compared to 35% (n = 27/71) in the group of patients that did not have an IDC; however, this difference did not reach statistical significance (P = 0.195, OR 0.57, 95% CI 0.25–1.33). For the period in which the study was carried out, our institution did not have restrictions on the use of carbapenems, it had an antimicrobial stewardship program under construction, but with poor administrative support and low human resources. There is enough evidence to support the importance and enormous impact that an Infectious Disease Service can have on the management of bloodstream infection [27], decreased mortality [28], lower healthcare costs and lower readmission rates [29].

In our study, FUBCs were taken in 21% of patients (n = 23) and 34% (n = 8/23) showed persistent bacterial growth. In the initial statistical analysis, it was observed that performing FUBCs behaved as a protective factor for the outcome of in-hospital mortality due to sepsis (OR 0.27, 95% CI 0.08–0.86, P = 0.021), however it did not reached statistical significance in logistic regression analysis. FUBCs appear to add little value in the management of gram-negative bacteremia [30], but recently, in a systematic review and meta-analysis by Thaden et al., it was found that positive FUBCs were associated with increased mortality in Gram-negative bacterial bloodstream infection relative to negative blood cultures (odds ratio, 2.27; 95% CI 1.54–3.34) [31].

## Limitations

This study had several limitations. First, the results were obtained retrospectively from a single center which may limit the generalisability of the results, however our findings are in line with other reported analyses. Second, most of the clinical and laboratory variables were based on the first measurements performed on each patient at the time of the initial presentation of sepsis, and whether there was variation in the laboratory variables, during the course of the disease, was not considered. Third, the analysis did not take into account the dose used and the rate of infusion of the antibiotic administered to each patient, factors that may be decisive in the outcomes according to pharmacokinetic and pharmacodynamic studies [32]. Fourth, the presence of ESβL was established by conventional laboratory methods and no genotypic confirmation was performed.

## Conclusions

Mortality after a bacteremic ESβL-E infection is very high. A low frequency of prescription of carbapenems in patients with severe clinical compromise, due to septic shock requiring vasoactive support and Pitt bacteremia score > 3 were identified as risk factors for in-hospital death. These findings have serious implications for antibiotic prescription. Involvement of an infectious disease specialist may help improve outcomes.

## Data Availability

The datasets used and/or analyzed during the current study are available from the corresponding author on reasonable request.

## Abbreviations

ESβL: Extended spectrum β-lactamases
ESβL-E: ESβL-producing Enterobacterales
CRBSI: Catheter-related blood stream infection
IQR: Interquartile ranges
COPD: Chronic obstructive pulmonary disease
CLSI: Clinical & Laboratory Standards Institute
MIC: Minimum inhibitory concentration
IDC: Infectious disease consultation
BC: Blood culture
FUBCs: Follow-up blood cultures
HACEK: *Haemophilus* spp., *Actinobacillus actinomycetemcomitans*, *Cardiobacterium hominis*, *Eikenella corrodens*, *Kingella* spp.
